# Nerve growth factor and glutamate increase the density and expression of substance P-containing nerve fibers in healthy human masseter muscles

**DOI:** 10.1038/s41598-021-95229-7

**Published:** 2021-08-02

**Authors:** Abdelrahman M. Alhilou, Akiko Shimada, Camilla I. Svensson, Peter Svensson, Malin Ernberg, Brian E. Cairns, Nikolaos Christidis

**Affiliations:** 1 Department of Restorative Dentistry, College of Dentistry, Umm Al-Qura University, Makkah al Mukarramah, Saudi Arabia; 2 Division of Oral Diagnostics and Rehabilitation, Department of Dental Medicine, Karolinska Institutet, and Scandinavian Center for Orofacial Neurosciences (SCON), Box 4064, 141 04 Huddinge, Sweden; 3 Department of Geriatric Dentistry, Osaka Dental University, Osaka, Japan; 4 Department of Physiology and Pharmacology, Center for Molecular Medicine, Karolinska Institutet, Stockholm, Sweden; 5 Department of Dentistry and Oral Health, Aarhus University, and Center for Orofacial Neurosciences (SCON), Aarhus, Denmark; 6 Faculty of Pharmaceutical Sciences, University of British Columbia, Vancouver, Canada

**Keywords:** Neuroscience, Ion channels in the nervous system, Neurotrophic factors, Sensory processing

## Abstract

Nocifensive behavior induced by injection of glutamate or nerve growth factor (NGF) into rats masseter muscle is mediated, in part, through the activation of peripheral NMDA receptors. However, information is lacking about the mechanism that contributes to pain and sensitization induced by these substances in humans. Immunohistochemical analysis of microbiopsies obtained from human masseter muscle was used to investigate if injection of glutamate into the NGF-sensitized masseter muscle alters the density or expression of the NMDA receptor subtype 2B (NR2B) or NGF by putative sensory afferent (that express SP) fibers. The relationship between expression and pain characteristics was also examined. NGF and glutamate administration increased the density and expression of NR2B and NGF by muscle putative sensory afferent fibers (P < 0.050). This increase in expression was greater in women than in men (P < 0.050). Expression of NR2B receptors by putative sensory afferent fibers was positively correlated with pain characteristics. Results suggest that increased expression of peripheral NMDA receptors partly contributes to the increased pain and sensitivity induced by intramuscular injection of NGF and glutamate in healthy humans; a model of myofascial temporomandibular disorder (TMD) pain. Whether a similar increase in peripheral NMDA expression occurs in patients with painful TMDs warrants further investigation.

## Introduction

Masticatory muscle pain (myalgia/myofascial pain) is one of the most prevalent symptoms among temporomandibular disorders (TMD)^1^. TMD-myalgia is localized muscle pain that is exacerbated by jaw muscle function and palpation^2^. This type of chronic pain is more common in women than men^3,4^. The pathophysiological mechanism involved in TMD-myalgia as well as the sex-related differences are not yet well understood. To investigate this aspect and to develop effective treatments for painful TMDs, standardized experimental pain models that mimic the clinical characteristics of this disorder are required^5–7^. For TMD-myalgia, several exogenous experimental pain models that use injections of different substances such as glutamate, serotonin, acidic saline, hypertonic saline and nerve growth factors (NGF) have been proposed^8–14^. Both glutamate and NGF have advantages over other pain models where experimental injection of these substances alone or in combination into healthy human masseter muscle produced similar pain characteristics involved in TMD myalgia^7,15,16^. However, the cellular and molecular mechanisms involved in these models are still not well addressed in the literature, especially for humans.

Substance P (SP) and NGF are important neurotransmitters/modulators that have been shown to mediate inflammatory hyperalgesia^17,18^. During inflammation NGF, by the help of cytokines, is thought to be released from different cells^19–23^. NGF binds to tyrosine kinase A (TrkA) receptors on nociceptive endings^24^, which in turn is retrogradely transported from the site of inflammation to the dorsal root ganglion leading to increased SP transcription^25^. In rats, experimentally induced inflammation of the gastrocnemius and soleus muscle increased the density of nerve fibers (mainly perivascular fibers) expressing SP or NGF^26^. However, administration of NGF alone has been shown to induce sensory hypersensitivity without any inflammatory response^27^. Therefore, it is of interest to know if these neurotransmitters also are involved in experimentally induced masseter myalgia. Especially since the majority of TMD does not show signs of inflammatory changes^28,29^.

Myalgia can be initiated by noxious stimuli (mechanical or chemical) applied to the muscle, which in turn can activate specific receptors in the muscle nerve fibers as a consequence of the elevation of algogenic substances, such as glutamate^30^. In patients suffering from TMD-related myalgia, the glutamate level is elevated in the masseter muscle interstitial fluid as well as in the saliva and plasma^31,32^. In rats, injection of either NGF or glutamate induces muscle sensitization, in part, through the activation of peripheral N-methyl-d-aspartate (NMDA) receptors (glutamate receptors)^8,11,12,33^. While these findings in animals suggest an interaction between NGF and NMDA receptors, their interaction in human muscle is still unknown. Therefore, this study aimed to investigate the effect of NGF and glutamate injections on the density of nerve fibers in general and on the density of putative sensory afferent (that express SP) nerve fibers as well as their expression of NMDA-receptor subtype 2B (NR2B) and NGF in human masseter muscle, to correlate expression with pain characteristics, and to determine any possible sex-related differences in these effects of combined injections.

## Methods

### Participants

Advertisements were posted on the Aarhus University campus (Denmark) and on an internet-page (https://aucobe.sona-systems.com/). In total, 15 healthy women and 15 age-matched healthy men (mean(SD) 24(4) years of age) were recruited. Screening for TMD was accomplished by using the diagnostic criteria for TMD (DC/TMD)^2^. Exclusion criteria were TMD related pain, facial pain, palpatory tenderness, neurological disorder, inflammatory diseases, fibromyalgia, whiplash-associated disorders, neuropathic disorders or pregnancy. Participants were informed not to use anti-inflammatory or analgesic medication for at least 24 h before the procedures and until the end of the experiment. All participants gave informed consent. The experiment followed the guidelines of the Helsinki declaration and was approved by the ethical committee in Aarhus, Demark (Midtjylland, approval No. 1-10-72-199-15).

### Study design

**Figure 1:**
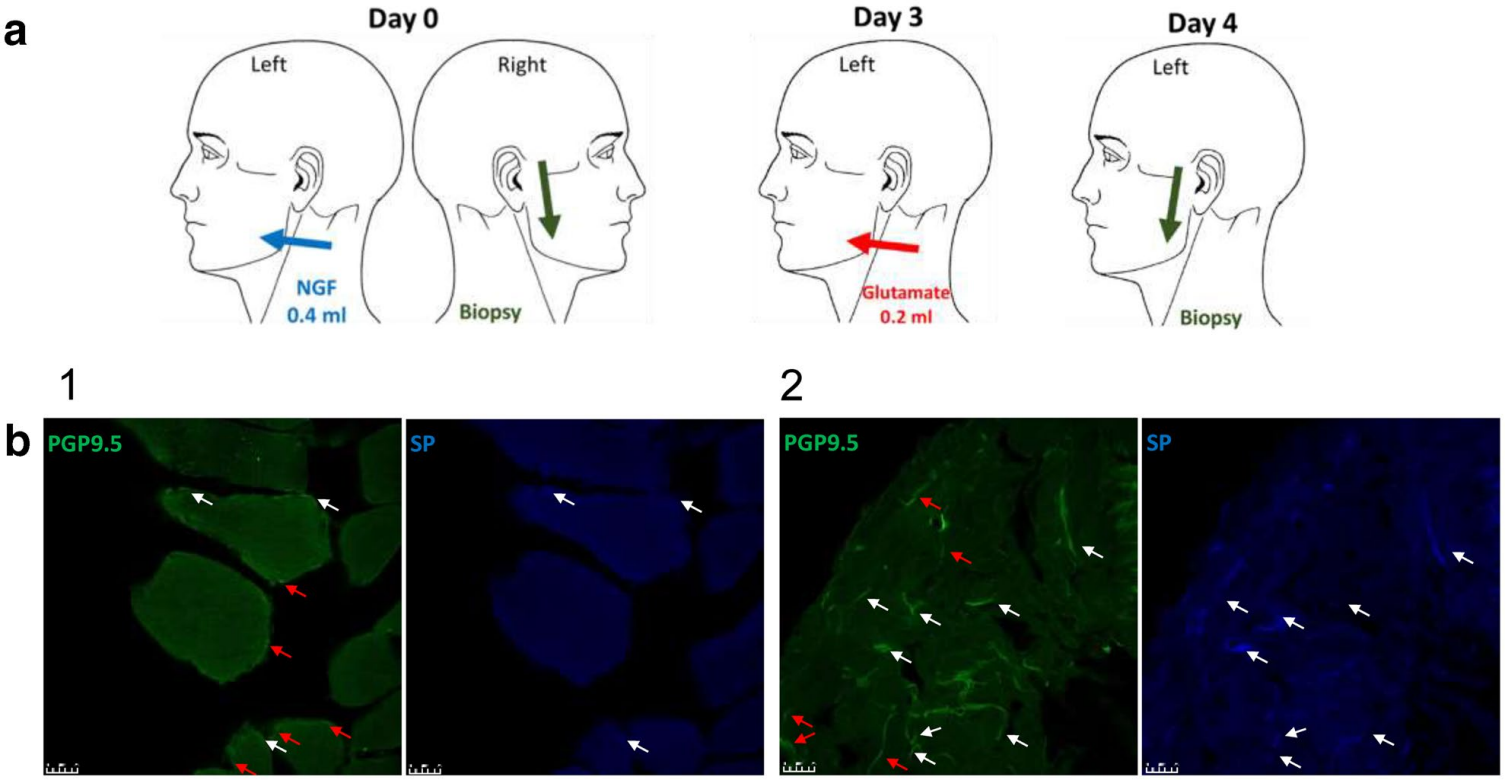
Figure displaying the methodology of the study. (**a**) Glutamate was injected three days after NGF into the left masseter muscle. One day after injection, a microbiopsy was taken from the injection site. (**b**) The left photomicrograph shows example image of PGP9.5 staining (green) while the right one shows SP expressing nerve fibers (blue) (1) associated with myocytes and (2) within connective tissue from one female participant on day 4. The white arrows indicate positive PGP 9.5 immunofluorescence signals co-expressed with positive SP signals, while the red arrows indicate positive PGP 9.5 signals without SP co-expression. Green: PGP 9.5, Blue: SP, Scale bar = 25 µm.

The study comprised three sessions (day 0, day 3, and day 4). On day 0, 0.4 mL NGF (25 μg/mL sterile solution; Skanderborg Apotek, Aarhus, Denmark) was injected into the masseter muscle on the experimental side (left side). On day 3, glutamate (1 M, 0.2 mL sterile solution; Skanderborg Apotek, Aarhus, Denmark) was injected into the masseter on the same side. The solution was previously tested to have its effect on the nerve fibers through the activation of peripheral NMDA receptors and not due to its hypertonicity^8^. The technique followed for injections (glutamate or NGF) was standard and precise as previously described^34^. Aqueous solution was used as a solvent for both injections. Masseter microbiopsies were obtained both on day 0 from the control side and on day 4 from the experimental (injection) side as has been previously described in detail^35,36^. In each session, pressure pain threshold (PPT), chewing-evoked pain and temporal summation pain were recorded from both sides before (e.g. baseline) and 5 min after the injections. Pain intensity at rest was recorded directly after injections on day 0 and 3 (Fig. 1a). The results from these examinations as well as DC/TMD results have been presented elsewhere^15^.

### Immunohistochemistry and image analysis

Biopsy samples were fixed over-night at 4 °C, with 4% paraformaldehyde. Prior freezing (− 80 °C), samples were rinsed with phosphate-buffered saline (PBS) and dehydrated first with a 20% and then with a 40% sucrose solution. Sliced sections were treated with 10% normal donkey serum in PBS for 1 h, and then incubated for 24 h with primary antibodies against *PGP 9.5* (1:250, anti-human mouse monoclonal, ABCAM Inc, Cambridge, England, ab72911), *NR2B* (1:200, anti-human rabbit polyclonal, ABCAM Inc, Cambridge, England, ab65783), *SP* **(**1:1000, anti-human guinea pig polyclonal, ABCAM Inc, Cambridge, England, ab10353), and *NGF* (1:20, anti-human goat polyclonal, R&D Systems Inc, 614 McKineley PL NE Minneapolis, AF-256-NA). Alexa 488 donkey anti-mouse, Alexa Fluor 546 donkey anti-rabbit, Alexa Fluor 633 donkey anti-goat (ThermoFisher, Burlington, ON, Canada), and Alexa Fluor405 donkey anti-guinea pig (Sigma-Aldrich, MO, USA) at a concentration of 1:700 were used as corresponding fluorescent secondary antibodies for PGP 9.5, NR2B, NGF and SP, respectively. The antibody specificity was checked by removing the primary antibody. Images for the stained sections were taken by a Leica TCS SPE Confocal Microscope (Leica microsystems, Wetzlar, Germany). The analysis was performed blindly by a researcher who did not participate in biopsy collection or coding. To distinguish and count PGP 9.5 positive nerve fibers and to measure their area, the analysis and image processing program known as ImageJ (Image Processing and Analysis in Java; National Institutes of Health, USA) was used. The program was also used to identify nerve fibers (PGP9.5) co-expression with other molecules (SP, NR2B and NGF)^36^. Fibers were recognized as positive whenever PGP9.5 fluorescent signals were greater than + 2 standard deviations (SDs) above the mean background of the image, and had a minimum width and length of 4 μm^11^. PGP 9.5 positive nerve fibers were found in different tissues within the muscle; however, the current study looked only at the nerve fibers that were associated with myocytes and connective tissue. Hematoxylin staining was performed to facilitate description of cell types within the biopsies. Groups of tubular or round well-defined cells with multiple nuclei at the periphery were regarded as myocytes^37^, while irregular tissue containing many cells and loose or dense fibers surrounding the myocytes was considered connective tissue^38^. Nerve fibers expressing SP were regarded as putative sensory afferent nerve fibers (Fig. 1b). The following formulas were used to calculate the density and expression frequency. Density equals the number of positive fibers in a tissue divided by the total area of the same tissue on an image and averaged over the number of images per subject. Expression frequency equals the number of PGP9.5 positive fibers that were co-expressed with other substances divided by the total number of PGP9.5 positive fibers in the image and averaged over the number of images for the subject. Specific details on image analysis using Image J programme are previously explained in another study by the authors^36^.

### Statistical analysis

Thirty participants were sufficient to test the hypothesis at a significant level of 0.05 and power of 0.80, showing an estimated difference of 30% and a standard deviation of 50% in nerve fiber expression. Moreover, it has been proved that groups of 12 or more are enough to detect significant sex differences related to experimental pain models^39,40^. When data were compared between days, only data from participants whose biopsies carried the same tissue (connective tissue or myocytes) on both days were included in the analysis. However, when data were analyzed for correlations, all actual data from participants were included (Table 1). Significant differences and interaction between factors (sex and day) in the density and expression frequency of nerve fibers were detected by using a parametric 2-way RM ANOVA test; this was followed by posthoc comparisons using the Bonferroni-test. The Pearson or Spearman correlations (depending on whether data was normally distributed) were used to examine the relationship between the peak pain intensity (5 min after glutamate injection on day 3) and the expression of SP, NGF and NR2B alone or their co-expressions (SP with NGF, SP with NR2B, NGF with NR2B, and all together) by nerve fibers on day 4, and also to test the relationship between the change (day 3 post glutamate injection/day 0 baseline) in mechanical pain characteristics and the expression of SP, NGF and NR2B on day 4. The level of significance was set to P < 0.05. For statistical analysis, the SigmaPlot for Windows version 14.0 software (Systat Software Inc., San Jose, CA, USA) was used.

**Table 1:** The number of participants.

	Men (n = 15)	Women (n = 15)
**Same tissue both days**		
Myocytes	8	8
Connective tissue	10	14
**Tissue day 0**		
Myocytes	8	8
Connective tissue	12	14
**Tissue day 4**		
Myocytes	10	13
Connective tissue	13	15

The table presents the number of participants whose biopsies contained myocytes and connective tissue on both days as well as the number of participants whose biopsies contained myocytes and connective tissue on day 0 and day 4.

## Results

The results of the combined effect of NGF and glutamate on the density of nerve fibers and expression of receptors and neuropeptides are presented. The difference between connective tissue and myocytes on density as well as on nerve fibers expression has been presented elsewhere^36^.

### The combined effect of NGF and glutamate on the density of nerve fibers

The average density of PGP 9.5 positive nerve fibers associated with myocytes did not differ between days (F = 0.842, P = 0.374) or sex (F = 0.063, P = 0.805). However, the density of PGP 9.5 positive nerve fibers expressing SP was significantly greater on day 4 compared to day 0 (F = 6.970, P = 0.019). No sex-related differences were detected (F = 1.459, P = 0.247). No significant interaction was found between factors (F = 0.078, P = 0.783).

There were no significant differences in the average density of PGP 9.5 positive nerve fibers between days (F = 0.494, P = 0.489) or between sexes (F = 0.100, P = 0.754) within connective tissue. No significant differences in the average density of PGP 9.5 positive nerve fibers expressing SP between days (F = 0.871, P = 0.361) or between sexes (F = 1.504, P = 0.233) were identified.

### The combined effect of injections on the expression of receptors and neuropeptides

#### Myocytes

Analyzed data from all participants showed an increase in the frequency of nerve fiber expression of SP alone (F = 13.713, P = 0.002), with NR2B (F = 10.599, P = 0.006) and with NGF (F = 5.151, P = 0.040) as well as all three markers together (F = 4.774, P = 0.046) on day 4 compared to day 0. No significant differences were detected between days in nerve fiber expression of NR2B or NGF alone or both of them in combination (Table 2). There were no significant differences in the expression frequency of SP, NR2B, or NGF between sexes. However, within day 4, the frequency of nerve fibers expressing SP alone (P = 0.032) was significantly higher in women compared with men (Table 2).

**Table 2:** The frequency (%) expression of nerve fibers associated with myocytes.

	All participants		Men		Women	
	Day 0	Day 4	Day 0	Day 4	Day 0	Day 4
SP	17 (12)	29 (10)^##^	14 (10)	23 (10)	20 (14)	35 (6)^#^*
NR2B	89 (17)	88 (9)	93 (3)	91 (5)	85 (24)	85 (11)
NGF	81 (24)	84 (15)	88 (6)	88 (6)	74 (33)	79 (21)
SP with NR2B	16 (12)	27 (10)^##^	13 (9)	22 (10)	19 (14)	32 (7)^#^
SP with NGF	16 (12)	25 (9)^##^	13 (10)	21 (9)	19 (13)	29 (8)
NR2B with NGF	78 (22)	79 (15)	85 (4)	84 (8)	72 (31)	74 (19)
ALL	15 (11)	24 (8)^##^	12 (10)	21 (9)	19 (13)	28 (7)

The table presents the mean (SD) frequency of nerve fibers expressing markers alone and co-expressions (SP with NR2B, SP with NGF, NR2B with NGF, or All), from all participants, men, women on day 0 and 4. All = SP with NR2B and NGF.

^##^Significant differences between days in all participants (2-way RM ANOVA; P < 0.05).

^#^Significant differences between days (Bonferroni; P < 0.05) within men or women.

*Significant differences between men and women (Bonferroni; P < 0.05) within day 0 or day 4.

#### Connective tissue

When data from all participants were used for analysis, no significant changes in the frequency of expression of SP, NR2B, or NGF by nerve fibers were detected between days (Table 3). However, the nerve fiber expression of SP (F = 6.296, P = 0.020), NR2B (F = 4.956, P = 0.037), SP with NR2B (F = 8.366, P = 0.008), SP with NGF (F = 4.375, P = 0.048) and all three markers together (F = 4.716, P = 0.041) was significantly higher in women when compared with men.

**Table 3:** The frequency (%) expression of nerve fibers within connective tissue.

	All participants		Men		Women	
	Day 0	Day 4	Day 0	Day 4	Day 0	Day 4
SP	61 (14)	58 (17)	56 (14)	48 (19)	64 (13)	64 (13)*
NR2B	57 (21)	62 (19)	48 (15)	53 (13)	64 (23)*	69 (20)
NGF	26 (19)	27 (13)	25 (17)	24 (13)	27 (20)	29 (13)
SP with NR2B	38 (19)	40 (18)	27 (11)	29 (18)	45 (20)*	47 (15)*
SP with NGF	14 (10)	16 (10)	11 (7)	11 (6)	16 (11)	19 (11)
NR2B with NGF	22 (18)	25 (13)	20 (14)	22 (12)	24 (21)	27 (13)
ALL	13 (9)	15 (9)	9 (7)	10 (6)	15 (10)	18 (12)

The table presents the mean (SD) frequency of nerve fibers expressing markers alone and co-expressions (SP with NR2B, SP with NGF, NR2B with NGF, or All), from all participants, men, women on day 0 and 4. All = SP with NR2B and NGF.

*Significant differences between men and women (Bonferroni; P < 0.05) within day 0 or day 4.

### The correlation between the expression of putative sensory afferent nerve fibers and mechanical sensitivity induced by NGF and glutamate

#### Myocytes

There was a significant positive correlation between the nerve fiber expression of SP alone and the percentage change in temporal summation pain (day 3 post glutamate injection/day 0 baseline) (r = 0.415, n = 23, P = 0.048). The nerve fiber expression of SP in combination with NR2B was also correlated with the percentage change in temporal summation pain (r = 0.437, n = 23, P = 0.036). No significant correlation was found for other mechanical sensitivity parameters (PPT and chewing-evoked pain) (P > 0.05).

#### Connective tissue

**Figure 2:**
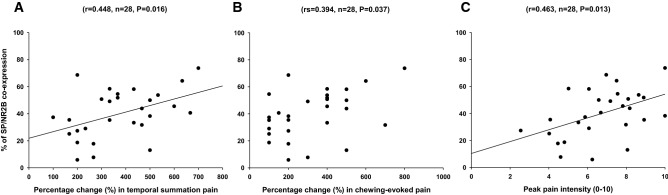
The scatter plot illustrates correlations between different pain characteristics and NR2B receptor expression by putative sensory afferent nerve fibers. The correlation between the co-expression of SP with NR2B by nerve fibers and the percentage change (day 3 post glutamate injection/day 0 baseline) in (**A**) temporal summation pain and (**B**) chewing-evoked pain. (**C**) is presenting the correlation of SP with NR2B co-expression with the peak pain intensity induced by injection of glutamate three days after NGF administration. *r* Pearson, *rs* Spearman correlation coefficient.

Data from all participants showed significant positive correlations between the expression of SP with NR2B by nerve fibers on day 4 and the percentage change (day 3 post glutamate injection/day 0 baseline) in temporal summation pain (r = 0.448, n = 28, P = 0.016) (Fig. 2A) as well as chewing-evoked pain (r_s_ = 0.394, n = 28, P = 0.037) (Fig. 2B). No significant correlations were found for PPT.

### The correlation between the expression of SP with NR2B and pain induced by glutamate

#### Myocytes

No significant correlations were found.

#### Connective tissue

A significant positive correlation was detected between the nerve fiber expression of SP with NR2B and the peak glutamate-evoked pain intensity in all participants (r = 0.463, n = 28, P = 0.013) (Fig. 2C). However, no significant correlation was found when data analyzed from men (r = 0.315, n = 13, P = 0.294) and women (r = 0.495, n = 15, P = 0.060) were undertaken separately.

## Discussion

The main findings of the current study were that: (1) the combination of NGF and glutamate injection into the masseter muscle increases the density and expression of putative sensory afferent nerve fibers that express SP; (2) the density of putative sensory afferent nerve fibers does not differ between sexes, but the expression of putative sensory afferent nerve fibers is greater in women than men. (3) The increase in temporal summation pain and chewing-evoked pain induced by NGF and glutamate is positively correlated with the expression of NR2B by putative sensory nerve fibers; and (4) peak glutamate-induced pain intensity is positively correlated with expression of NR2B by putative sensory nerve fibers in NGF-sensitized masseter muscle.

SP is an important peptide that is involved in many physiological and pathophysiological processes, including nociception^41^. At the level of the trigeminal ganglion, SP was mostly found in unmyelinated small diameter nerve fibers^42^ which comprise 10–30% of all trigeminal nerve fibers^43^. SP expressing sensory afferent nerve fibers project to the brainstem trigeminal sensory nucleus, and upon release increase the excitability of trigeminal sensory neurons^44,45^ Injury induced by extraction of maxillary molars increases the number of SP immunoreactive neurons in the trigeminal ganglion^46^. Moreover, NGF increases the expression of SP by masseter ganglion neurons in female rats^11^. In rats, it has been shown that NGF can increase the level of SP at the dorsal root ganglion^49^. Thus, NGF contributes to central sensitization partly by increasing the release of neuropeptides including SP^47^. The current study is the first, to our knowledge, to show an increase in the frequency of nerve fiber expression of SP as a consequence of NGF and glutamate injections into human muscles. This finding may suggest the involvement of this nociceptive peptide in peripheral sensitization. However, a recent published article has demonstrated lack of changes of plasma SP levels in patients with TMD compared to healthy controls^32^. Moreover, injection of SP into the temporalis muscle was not painful, and into the tibialis muscle failed to produce hyperalgesia to PPT^48,49^. Large-scale trials targeting SP-antagonism in humans failed to successfully reduce pain^50^. These contradictory results could be attributed to differences between rats and humans in terms of the contribution of SP to pain mechanisms, as in humans, SP can contribute to other biological processes such as vasodilatation and inflammation^51,52^. It is still possible that SP acts to enhance glutamate-induced mechanical sensitization of masticatory muscle in humans^49,53^.

There is some evidence which suggests that NGF modulates both the expression and function of NMDA receptors to alter the response properties of masseter muscle sensory afferent nerve fibers. NGF injection into the masseter muscle of healthy participants induced mechanical sensitization that lasted up to 3 weeks^13,16^. In rats, there is evidence that NGF-induced masseter muscle sensitization is caused by increased expression of NMDA receptors by sensory afferent fibers^11^. Injection of glutamate can also evoke masseter muscle sensitization through the activation of peripheral NMDA receptors^12,54^. Injection of glutamate into masseter muscles pretreated with NGF failed to cause an increase or decrease in PPT^15^, which could be an indication that glutamate and NGF can share a common pathway, thus preventing additional sensitization when these substances are used together^13^. It is also possible that a ceiling effect occurred by NGF on PPT and that additional sensitization by glutamate, thus was not possible. The current study showed that experimental myalgia induced by NGF and glutamate can increase the density of masseter sensory afferent nerve fibers as well as the expression of NR2B and NGF. This finding supports the suggested interaction of NGF and NMDA receptors^11^ and may indicate their involvement in the peripheral mechanism that underlies muscle sensitization.

Earlier studies showed that an injection of NGF into the masseter muscle reduced PPT more in women than in men^16,55^. The reduction of PPT in women was associated with an increased expression of NMDA-receptors by sensory afferent nerve fibers^56^. It has also been reported that injection of glutamate into the masseter muscle produces more intense pain in women than in men^40,57^. In female rats, increased estrogen levels are associated with higher expression of NR2B subunit-containing NMDA-receptors by masseter ganglion neurons and an increased sensory afferent nerve fiber discharge evoked by injection of NMDA into the masseter muscle^54^. NGF-induced mechanical sensitization was suggested to be due to the greater co-expression of SP with NR2B in masseter ganglion neurons of female as compared with male rats^11^. Consistent with animal reports, the current study showed sex differences related to the putative sensory afferent nerve fiber expression of NR2B subunit-containing NMDA-receptors as well as NGF. Hence, one can speculate that the sex-related differences in NGF induced muscle sensitization and glutamate evoked muscle pain are similar in rats and humans, and thereby also attributable to the sensory afferent nerve fiber expression of NR2B and NGF. However, the present study found no association between the expression of NMDA-receptors by nerve fibers and the change in PPT in women. The lack of association in the current study compared to our previous study^56^, is not unexpected as no sex-related differences in masseter muscle sensitization were detected post injection of glutamate into the masseter muscle^8,40^.

In a previous study we have presented a positive correlation between peripheral nerve fibers expression of NMDA receptors and pain characteristics after glutamate injection into the masseter muscle^56^. The present experimental pain model also demonstrates a significant association between the nerve fiber expression of peripheral NMDA-receptors and pain characteristics (pain intensity, chewing-evoked pain and temporal summation pain) reminiscent of pain complaints in individuals with TMD myalgia. Masticatory muscle pain and fatigue induced by chewing are common signs and symptoms of TMD myalgia^58^. Peak pain intensity reported by healthy participants after injection of glutamate into the masseter muscle is similar to the peak pain reported by patients with TMD^7^. Temporal summation of fixed mechanical stimulation can reflect central sensitization in an experimental pain model, and has been shown to be dependent on the activation of NMDA receptors^59^. Temporal summation pain reported by patients with TMD was higher than in healthy controls^60,61^. These findings together strengthen the theory that NMDA-receptors expressed peripherally by sensory afferent fibers and/or in the central nervous system plays a role with regard to the pathogenesis of TMD myalgia^12,33,62^, and that increasing NMDA receptor expression may cause the muscle to be more sensitive to pain.

A possible limitation for this study is that the number of sensory afferent fibers within muscle tissues was likely underestimated, as the expression of other neuropeptides associated with sensory afferent fibers was not assessed. For example, calcitonin gene-related peptide (CGRP) is thought to play an important role in nociception^63^ and is highly expressed by masseter muscle sensory afferent nerve fibers in rats^11^. Indeed, co-expression of CGRP and NMDA receptors was also increased in female rats after masseter muscle injection of NGF. If NGF exerts a similar effect on these sensory fibers in humans, then our study also likely underestimated the increase in NMDA expression that could be induced by NGF. Unfortunately, at the time this study was conducted, there was no available CGRP antibody compatible with the NMDA and PGP 9.5 antibodies for humans. Future studies will be required to address this question.

## Conclusions

Injections of NGF or glutamate are useful experimental models for the investigation of TMD myalgia^16^. The current study has demonstrated that cellular and molecular changes occur after combined injection of glutamate and NGF into the human masseter muscle. The combined injections increased the density of SP expressing nerve fibers as well as the expression of NMDA receptors and NGF by putative sensory afferent nerve fibers. This increase in expression was significantly greater in women than in men. Further, the expression of NMDA-receptors by putative sensory afferent nerve fibers was significantly associated with increased pain during functional activity and increased muscle pain sensitivity in both men and women. Hence, and in accordance with previous animal findings^11,12^ the current human study can conclude that NMDA-receptors and NGF expressed by peripheral putative sensory afferent nerve fibers seem to play an important role in the mechanisms of muscle sensitization and sex-related differences in pain reports associated with this model of masseter muscle myalgia. However, whether these results are relevant to the mechanisms of clinical muscle pain, for example, in myofascial TMD, is an open question that warrants further investigation.

## Data Availability

The datasets generated during and/or analyzed during the current study are available from the corresponding author on reasonable request.
